# A qualitative study exploring the determinants of maternal health service uptake in post-conflict Burundi and Northern Uganda

**DOI:** 10.1186/s12884-015-0449-8

**Published:** 2015-02-05

**Authors:** Primus Che Chi, Patience Bulage, Henrik Urdal, Johanne Sundby

**Affiliations:** Peace Research Institute Oslo, PO Box 9229, Grønland Oslo, Norway; Institute of Health and Society, University of Oslo, PO Box 1130, Blindern Oslo, Norway; International Organization for Migration, Plot 6A, Naguru Crescent, Kampala, Uganda

**Keywords:** Maternal health, Determinants, Post-conflict, Service utilisation

## Abstract

**Background:**

Armed conflict has been described as an important contributor to the social determinants of health and a driver of health inequity, including maternal health. These conflicts may severely reduce access to maternal health services and, as a consequence, lead to poor maternal health outcomes for a period extending beyond the conflict itself. As such, understanding how maternal health-seeking behaviour and utilisation of maternal health services can be improved in post-conflict societies is of crucial importance. This study aims to explore the determinants (barriers and facilitators) of women’s uptake of maternal, sexual and reproductive health services (MSRHS) in two post-conflict settings in sub-Saharan Africa; Burundi and Northern Uganda, and how uptake is affected by exposure to armed conflict.

**Methods:**

This is a qualitative study that utilised in-depth interviews and focus group discussions (FGDs) for data collection. One hundred and fifteen participants took part in the interviews and FGDs across the two study settings. Participants were women of reproductive age, local health providers and staff of non-governmental organizations. Issues explored included the factors affecting women’s utilisation of a range of MSRHS vis-à-vis conflict exposure. The framework method, making use of both inductive and deductive approaches, was used for analyzing the data.

**Results:**

A complex and inter-related set of factors affect women’s utilisation of MSRHS in post-conflict settings. Exposure to armed conflict affects women’s utilisation of these services mainly through impeding women’s health seeking behaviour and community perception of health services. The factors identified cut across the individual, socio-cultural, and political and health system spheres, and the main determinants include women’s fear of developing pregnancy-related complications, status of women empowerment and support at the household and community levels, removal of user-fees, proximity to the health facility, and attitude of health providers.

**Conclusions:**

Improving women’s uptake of MSRHS in post-conflict settings requires health system strengthening initiatives that address the barriers across the individual, socio-cultural, and political and health system spheres. While addressing financial barriers to access is crucial, attention should be paid to non-financial barriers as well. The goal should be to develop an equitable and sustainable health system.

**Electronic supplementary material:**

The online version of this article (doi:10.1186/s12884-015-0449-8) contains supplementary material, which is available to authorized users.

## Background

Although the 2013 UN Millennium Development Goals (MDGs) progress report shows that many regions of the world have made progress on the fifth goal of improving maternal health, the region of sub-Saharan Africa (SSA) is still lagging behind, and will not be able to meet the agreed targets of ‘reducing by three quarters, between 1990 and 2015, the maternal mortality ratio’ and ‘achieving, by 2015, universal access to reproductive health’ [1]. Within SSA, countries in or emerging from armed conflicts are among the hardest hit. The deteriorating impact of armed conflict on maternal health is well acknowledged, and tends to linger even after the end of the conflict [2-4]. Armed conflicts are associated with higher total fertility and maternal mortality rates [5]. A 2010 review [6] of maternal mortality in 181 countries spanning 1980–2008 revealed that in 2008, 50% of all maternal deaths occurred in only six countries (India, Nigeria, Pakistan, Afghanistan, Ethiopia, and the Democratic Republic of the Congo); all of which have experienced recent armed conflict. For over a decade, the 10 countries ranked lowest on the *Save the Children’s* ‘State of the World’s Mothers Index’ have been conflict and post-conflict states [7]. Similarly, the 10 countries ranked lowest in the UN Human Development Index for the last decade are either in conflict or emerging from conflict. In this regard, armed conflict has been described as an important contributor to the social determinants of health [8-10] and a driver of health inequity [11]. Armed conflicts tend to limit access to maternal, sexual and reproductive health services (MSRHS) due to high levels of insecurity and high opportunity costs of accessing such services.

The uptake of MSRHS is closely associated with improvements in maternal health. For instance, quality antenatal care (ANC) should optimally reduce the risk of poor pregnancy outcomes, and a caesarean section can be obtained only when a woman seeks care at a health facility. To enhance women’s utilisation of these health services in post-conflict societies, an important step will be to explore the factors that may hinder and facilitate their uptake of services in these contexts. While much work has been done on the determinants of maternal health utilisation [12-16] including demographic, socio-economic, cultural, and health related factors, a general conclusion appears to be that the importance and impact of the factors varies from one setting to another. With health systems in conflict and post-conflict countries faced with challenges such as damaged infrastructure, limited human resources, weak stewardship and a proliferation of non-governmental organisations without proper coordination, this results in the delivery of disrupted and fragmented health services [17]. Hence, the utilisation of MSRHS is likely to be affected.

Burundi and Uganda are among the countries in Sub-Saharan Africa that are not poised to meet the fifth MDG goal of improving maternal health. They have both experienced brutal civil wars that claimed tens of thousands [18] of lives and displaced millions of people. Burundi experienced an ethnic conflict from 1993–2005 that led to the displacement of approximately 1.2 million people [19]. Although the country has been experiencing some gradual improvements in general population health, the population life expectancy stands at 53.9 years, with one of the highest maternal mortality ratios (800 deaths per 100,000 live births) and total fertility rates (6.1) in the world (UN World Fertility Patterns 2013; UN MDG indicator monitoring database). The Northern region of Uganda is recovering from over 20 years of armed conflict between the Lord’s Resistance Army and the Ugandan Government that resulted in the disruption of health services, massive population displacement and erosion of traditional and family structures [20]. The number of people displaced by the conflict was estimated at 2 million [17,20]. With a total fertility rate of 6.3, the Northern region ranks the highest in the country, with a median age at first birth of 17.8 years [21]. Uganda has a life expectancy of 59 years and maternal mortality ratio of 310 per 100,000 live births (UN World Fertility Patterns 2013; UN MDG indicator monitoring database), and the corresponding data for the Northern region might be worse.

The health system in Burundi is organized as a pyramid structure with three levels, comprising the central, intermediate and peripheral levels. The central level involves the Office of the Minister with its associated directorates, departments, programmes and related services, and it is responsible for formulating sector policy, strategic planning, coordination, mobilization and allocation of resources and oversight-evaluation. The intermediate level is comprised of 17 provincial health bureaus, in charge of coordinating all health activities of the province, supporting the health districts and ensuring proper collaboration between sectors. The peripheral level is responsible for the delivery of healthcare, and as of 2010 it was comprised of 45 health districts, including 63 hospitals and 735 health centres (423 public, 105 approved religious facilities and 207 private facilities) distributed throughout the 129 cities in the country [22]. All health centres are expected to offer a minimum package of services, including treatment and prevention consultation services, laboratory, pharmacy, health promotion and health education services, as well as in-patient observation. However, a recent survey found that 45% of health centres were unable to provide the complete recommended minimum package due to lack of personnel, infrastructure, equipment or medication [22]. For example, the survey reported that the physician-to-resident, and midwife-to-woman of child bearing age ratios are 1 per 19,231 (WHO recommended ratio is 1 per 10,000) and 1 per 123,312 (WHO recommended ratio is 1 per 5,000), respectively. Furthermore, a 2010 national survey of emergency obstetric and neonatal care (EmONC) facilities found that only five health centres were offering the recommended basic EmONC services, while 17 hospitals could provide comprehensive EmONC services – with the latter having a poor geographical distribution nationally [22]. These are recurrent challenges that appear to be happening against the backdrop of low government expenditure on health, as shown in Table 1. The current health situation in Burundi is described as precarious, with a fragile health system characterized by a high burden of communicable and non-communicable diseases, particularly affecting pregnant women and children [23]. According to the 2009 statistics, the diseases that were the primary causes of morbidity and mortality were malaria, acute respiratory infections, diarrheic diseases, malnutrition, HIV/AIDS and tuberculosis [22]. Following the end of the armed conflict in 2007, Burundi has been gradually restructuring the health system, with the introduction of the district health system to implement primary health care, coupled with the implementation of a performance-based financing (PBF) programme [24]. Furthermore, the government has introduced a free health care policy for pregnant women and children under 5, and a health insurance scheme for the informal sector. With these reforms in place, it is estimated that about 50% of the population (mainly pregnant women and under-fives) have universal access to health care [23]. The reforms have equally led to an increase in the use of health services, better quality of treatment, and a greater number of health personnel in rural areas [23].

**Table 1:** **Health system indicators in Burundi and Uganda**

**Indicators**	**Countries**	
	**Uganda**	**Burundi**
Density of physicians per 1,000 population	0.12 (2005)	0.03 (2004)
Density of nurses and midwives per 1,000 population	1.31 (2005)	0.06 (2004)
Total expenditure on health as % of GDP (2011)	9.5	8.7
General government expenditure on health as % of total government expenditure (2011)^§^	10.8	8.1
Gender-related Development Index rank out of 148 countries (2012)	110	98
Human Development Index rank out of 186 countries (2012)	161	178

Source of data: Global Health Observatory – April 2014 (http://apps.who.int/gho/data/node.country.country). § = The WHO recommends that member states should spend at least 15% of their annual budget on health, ( ) = year of publication.

Uganda equally operates on the district health system model, with the decentralization of health service delivery to the health district and health sub-district levels. The delivery of healthcare is done by both public and private actors, with the government owning 2,242 health centres and 59 hospitals, compared to 613 health facilities and 46 hospitals run by private not-for-profit actors (PNFP), and 269 health centres and 8 hospitals run by private health practitioners as of 2010 [25]. A major proportion of the PNFP providers are faith-based religious organizations, including the Uganda Catholic, Protestant, Orthodox and Muslim Medical Bureaus. A minimum package of health services is provided at all levels of health care in both the public and private sectors. Since 2001, user fees have been abolished in all public health facilities, but utilisation of health services has been hampered by poor infrastructure, lack of medicines and other health supplies, shortage of health workers, and low salaries [25]. Furthermore, concerns around long waiting times, unofficial fees in public facilities, and poor attitudes among health workers have also limited the utilisation of health services [26]. The disease burden in the country is dominated by communicable diseases, with maternal and perinatal health conditions contributing to the high mortality [27]. While Uganda is experiencing a shortage of health workers (as highlighted in Table 1), following a recent government recruitment exercise, overall staffing levels at higher level health centres such as Health Centres IV and III has improved from 57% in 2012 to approximately 70% in 2013 [27].

**Figure 1:**
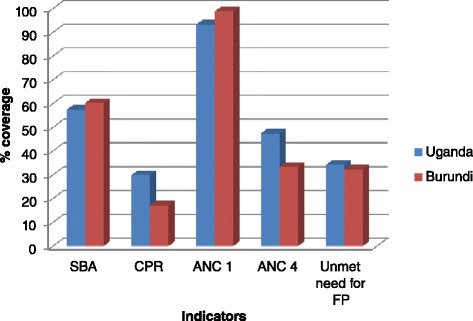
**Reproductive health outlook for Burundi and Uganda.** Source of data: UN MDG indicators monitoring database (http://mdgs.un.org/unsd/mdg/data.aspx). SBA: Skilled birth attendance; CPR: Current contraceptive use among married women 15–49 years old, any method; ANC 1: Antenatal care coverage, at least one visit; ANC 4: Antenatal care coverage, at least four visits; FP: Family planning.

Some key reproductive health indicators in Burundi and Uganda are displayed in Figure 1. While ANC coverage for at least one visit in both countries is quite satisfactory, the other health indicators such as contraceptive uptake, unmet need for family planning and ANC coverage for at least four visits are disappointing and require some improvement.

This study aims to explore the determinants of women’s utilisation of MSRHS in the post-conflict settings of Northern Uganda and Burundi and how exposure to armed conflict may affect these factors. Our main research question was ‘*what are the factors that encourage and discourage women’s uptake of maternal and reproductive health services and how does exposure to armed conflict affect these factors?*’ Through this study, we seek to contribute to the broader literature on determinants of maternal health and health-seeking behaviour in conflict and post-conflict settings.

## Methods

### Study settings

The study was undertaken in two provinces in Burundi (Bujumbura Marie and Ngozi) and a district in Northern Uganda (Gulu). In Burundi, participants were recruited from the cities of Bujumbura and Ngozi and the rural and semi-urban communes of Ruhororo in Ngozi Province and Kinama in Bujumbura Mairie province respectively. In Gulu district, the participants were recruited from the rural sub-counties of Koro, Bobi and Bungatira, and the municipality of Gulu, which comprises of four sub-counties. Maps of the study areas are found in Additional file 1.

### Data collection method

This is a qualitative study based on in-depth interviews (IDIs) and focus group discussions (FGDs). Interviews and FGDs were conducted in the local languages (Kirundi in Burundi and Luo in Northern Uganda), French or English (where applicable). All English interviews and FGDs were carried out by the principal investigator (PCC), while those in the local languages and French were conducted by trained local research assistants. The fieldwork took place from June until September 2013.

### Study participants

Study participants were recruited from staff members of local and international NGOs and local health providers (LHPs) working in the domain of maternal, sexual, and reproductive health (MSRH). The second group of participants consists of women of reproductive age, living in rural and semi-urban areas. Since we are interested in also capturing the effect the conflict had on MSRHS, NGOs and health providers invited to participate in the study had developed, supported and/or provided MSRHS during the conflict or shortly after the conflict. Similarly, the women we invited to participate in the study had sought or attempted to seek for such services as well during such periods.

### Issues discussed

The interviews and FGDs focused specifically on the general state of MSRH in Burundi and Northern Uganda, aimed at describing the general state of maternal health and understanding the factors affecting women’s utilisation of basic MSRHS, taking into consideration the possible effects of the recent conflict. The detailed guides for the interviews and FGDs for each of the participant categories can be found in Additional file 2. A sample of some of the questions posed to participants during the interviews and FGDs include:

1. What factors do you think affects women’s utilisation of health services during pregnancy and childbirth? (explore possible factors such as quality of care/treatment provided by health provider, costs for services, travel distance, lack of knowledge on when to seek care etc).
2. Have these factors changed over time? (probe to inquire how?).
3. Do you have any ideas how the past conflict might have affected this? (probe to inquire how was the use before and after etc).

### Ethical considerations

Ethics approval for the study was obtained from the Regional Committee for Medical and Health Research Ethics, South-East (Norway); le Comité National d’Ethique pour la Protection des êtres Humains Participant à la Recherche Biomédicale et Comportementale (Burundi); and Gulu University Institutional Review Committee (Uganda). We also received permission from local administrative and health authorities. All participants/informants gave their informed consent before participating in the study, and their anonymity, privacy and confidentiality was respected. Written or oral consent was acceptable and approved by the relevant ethics committees.

### Data management and analysis

All interviews and FGDs were audio-recorded and later transcribed and translated into English (where applicable). English transcripts were imported into the QRS Nvivo (QSR International, 2012). Considering the multidisciplinary nature of the research team and that the data were mainly made up of semi-structured interview transcripts, the framework method [28] was used to manage and analyze the data. Three team members open-coded the transcripts on Nvivo and Microsoft® Word. Microsoft® Word was used for coding and analysis by one of the co-authors who did not have access to Nvivo. The codes were descriptions or labels of specific ideas identified as the transcripts were read. Two team members reviewed the codes that were developed, and the inter-coder reliability was high. Inter-related or similar codes were then clustered into different categories, and the categories were subsequently grouped into specific themes. The themes were inductively and deductively developed. Inductive means that they were anticipated from the design of the interview and FGD guides and consciously explored in the interviews and FGDs. Deductive means that they were not anticipated during the design, but rather identified during the review of the transcripts. There was a constant interplay between data collection, analysis and theme development, with new and dominant ideas that emerged in earlier interviews and FGDs being explored deeper in subsequent and later interviews and discussions. The themes were also developed taking into consideration the main factors affecting women’s utilisation of maternal health services proposed by Wild et al.’s [29] multilayered explanatory model (i.e. individual, social, cultural, political and health system factors).

A detailed description of the methods is provided in Additional file 1.

## Results

### Characteristics of study participants

As shown in Table 2, we had 63 interviews and 8 FGDs across the study settings in Burundi and Northern Uganda. A total of 115 individuals participated in the study: 46 women of reproductive age (‘women’), 32 ‘LHPs’ and 37 NGO staff. The LHPs included those working at the facility (LHP) and senior administrative officials working at the local ministry of health (LHP-Policy maker). Within the NGO category we had three sub-categories of respondents: NGO, NGO-Health providers (NGOs that also provide health services) and NGO-Policy makers (mainly UN-based NGOs).

**Table 2:** **Number of interviews and FGDs, by study site and participant category**

**Country**	**Study areas**	**Participants/Informants**			**Total**
		**Women**	**LHPs**	**NGOs**	
Burundi	Bujumbura Marie and Ngozi provinces	11 Interviews & 2 FGDs	9 Interviews & 1 FGD	11 Interviews & 1 FGD	31 interviews & 4 FGDs
Uganda	Northern Uganda	10 Interviews & 2 FGD	12 Interviews & 1 FGD	10 Interviews & 1 FGD	32 interviews & 4 FGDs
**All countries**		21 interviews & 4 FGDs	21 interviews & 2 FGDs	21 interviews and 2 FGDs	**63** interviews & **8** FGDs

In the following paragraphs we present the participants’ perceived current status of MSRH and level of utilisation of MSRHS, and the determinants of women’s utilisation of these services vis-à-vis the possible effects of exposure to conflict. The individual determining factors were quantified by obtaining the percentage of participants within each of the categories that mentioned a specific factor during an interview or FGD.

#### Current status of maternal and reproductive health

Over two-thirds of the LHP and NGO respondents in both Northern Uganda and Burundi felt that the general status of MSRH is poor, but has been improving in the aftermath of the conflict. They mentioned positive evolution of some MSRH indicators such as maternal mortality, skilled attendance at birth and contraceptive uptake coupled with the initiation of some specialized services like cervical cancer screening as key pointers to improvements in maternal health.

“*During the time of the war maternal mortality was very high in this region. But currently it is between 300 and 400 per 100 000. But around that time it was around 600 to 700…*” NGO, FGD – Gulu, Northern Uganda

“[In Ngozi Province] *in 2005, the percentage of women who deliver at the health facility was 40 percent but now it is about 70 percent. The uptake of family planning in 2005 was 10 percent but now it is around 25 percent.*” LHP-Policy maker, IDI – Ngozi, Burundi

The positive observations made by the LHP and NGO respondents were also affirmed by the women, most of whom felt that the number of pregnant women from their communities attending ANC and delivering at the health facility had been increasing since the conflict ended. The increasing uptake of these services was largely associated with improved physical safety, an increase in the number of health facilities that has reduced the distance people have to travel to seek care, and an ongoing government health policy of free healthcare for all in government health facilities (for Uganda), and free healthcare for pregnant women and children under five years (for Burundi).

“*With the president’s law (*free health care for pregnant women and children under five*), things have evolved in a positive way. Death rate for pregnant women has reduced considerably…Today a death of a pregnant woman is considered as an accident.*” Woman, IDI – Kinama, Burundi

“*In the past it was very difficult to reach the hospital but now services are closer…If you compare the time that one would take to reach the hospital in the past, you will find that it is better now*” Woman, IDI – Bungatira, Northern Uganda

#### Determinants of women’s utilisation of MSRHS and the effect of conflict exposure

A combination of complex and inter-related factors affecting the utilisation of MSRHS by women were identified across the study sites. A number of these factors were associated with exposure to past conflict. Using the Wild et al. [29] multilayered person-centred exploratory model on the utilisation of maternal health services we grouped the factors into the following themes: individual, socio-cultural, and political/health system levels. Table S2 (Additional file 3) shows the main factors identified by the different categories of participants across the study sites. The perspectives of the LHP and NGO categories of respondents were highly similar, hence these were merged. The determinants were largely presented as ‘push’ (barrier) or ‘pull’ (facilitating) factors and included both supply and demand side factors. The factors identified are presented vis-à-vis the various participant categories.

### Individual level

#### Women

The most common individual level factor that encouraged women across the study sites to utilise MSRHS like family planning was the difficulty with catering for existing children. This factor was raised by over 80% of the women. This is because following the end of the conflict there has been a very strong cultural desire to replace family members lost during the conflict. The demand for family planning services was also facilitated by desire for women to recuperate after child birth, prevailing pressure on the existing limited land resources, and high incidence of land disputes following relocation of families back to their communities from internally displaced persons (IDP) camps as the insurgency ended. This has limited the quantity of food that can be cultivated.

“*In general, the living conditions are very difficult. You cannot give birth to too many children when you do not have something to give them. Nowadays, there is not enough space for those children. These are some of the reasons why women seek for family planning services*” Woman, IDI – Koro, Northern Uganda

Previous experience with or fear of a complicated or abnormal delivery and the development of an obstetric danger sign (as well as the severity of the manifestation of the sign) were also important individual level facilitating factors (76%). Most of these decisions tended to have been undertaken with the backdrop of little or no help with household chores for many of these women.

In Burundi, the desire to ensure that the newborn was registered and granted a birth certificate which gave free access to healthcare under the new targeted healthcare policy was a very strong ‘pull’ factor (90%) for facility delivery.

“*The reason why women are motivated to visit the health facility when pregnant is because they are afraid of delivering at home. When you deliver at home, your baby is not registered*.” Women, FGD - Ruhororo, Burundi

Normally, the birth notification document that is required to make a birth certificate is provided at the facility after delivery, hence women who do not deliver at the facility often struggle to have a birth certificate issued for their newborn. Other ‘pull’ factors that emerged included the desire to know their HIV status and to learn about the evolution of the pregnancy.

One main barrier identified across the sites, and especially in Northern Uganda, included past unpleasant experiences or fear of such experiences at the hands of health providers at the health facility, discouraging some women from seeking services (60%). With extensive impoverishment among the rural women who were temporarily displaced from their communities during the conflict, many of them felt despised, looked down upon, and poorly received by health personnel when visiting the health facility. Also, 43% of the women cited past experience of severe side effects of contraceptives, such as heavy bleeding and increase in weight, as a barrier to the uptake of modern contraceptives. In Burundi, approximately 20% of the women reported that some women were discouraged from seeking maternal health services for fear of being diagnosed with HIV infection. A few respondents mentioned the lack of ‘good clothes’ to wear as a barrier to facility delivery. Some who could not afford ‘good’ clothes preferred to deliver at home, especially within urban and semi-urban areas.

“*The things that discourage some are…lack of good clothes to wear in order to go to the hospital or health centre without being laughed at; lack of clothes for the newborn; and ashamed of being laughed at if they do not have something to eat whereas other patients have relatives to bring them good food*.” Woman, IDI - Kinama, Burundi

The educational level was also mentioned (24%) as an individual level determinant for women’s utilisation of MSRHS, with more educated women being more likely to seek these services. Lack of safety was identified as an important barrier to education during the conflict. Some respondents (41%) also felt that the high burden of domestic chores that some women have to undertake, ranging from cooking, cleaning, and farming, may discourage the use of facility-based health care.

“…*I think that it is because of the too much work that women have at home that stops them from going to the hospital.*” Woman, IDI – Bobi, Northern Uganda

#### LHPs and NGOs

Most of the individual level factors that the LHP and NGO respondents felt affected women’s utilisation of MSRHS were largely similar to those mentioned by the women themselves across the study settings. In Northern Uganda, the main facilitators mentioned *only* by LHPs and NGOs included availability of contraceptive methods that could be concealed from the male partners/husband (such as implants) (60%); and a deep sense of trust that their privacy and confidentially would be respected by the health providers (50%) – especially for HIV positive women, and for those secretly requesting family planning and post-abortion care services. The corresponding facilitators for Burundi included HIV positive women’s desire to protect their unborn child from HIV infection (70%); and realization of the importance of family planning – including personal positive experiences with contraceptive use (65%); improving knowledge; and understanding the evolution of their pregnancy. The barriers were similar across the participant categories in Northern Uganda, and many respondents (74%) in this participant category felt that the poor health-seeking behaviour of some women was due to the conflict-engendered low literacy levels among the population.

Barriers mentioned only by LHPs and NGOs in Burundi were ignorance of the importance of these services, lack of money for transport and medication, in some areas confidence in traditional birth attendants to undertake home deliveries, and personal religious convictions.

### Socio-cultural level

#### Women

The most common socio-cultural factors raised across the study sites were poverty (85%), community- and male-partner perceptions about modern contraceptives (80%), and the ease of reaching the health facility (70%), including the distance to the facility and the nature of the road network. These were to some extent associated with the conflict, as huge segments of the population, especially in rural areas, are still struggling to rebuild their livelihoods destroyed by the conflict. Infrastructure, including roads, schools and health facilities, was generally disrupted during the conflict. With respect to contraceptive uptake, rumours and myths about modern contraceptives, fear of side effects, and male-partner opposition to uptake were perceived as important barriers.

“*There are some women who do not believe the contraceptive methods because they think that these methods will prevent them from reproducing in the future*” Woman, IDI – Kinama, Burundi

“*Some say that family planning* [modern contraceptive] *is going to kill their eggs…While others think family planning can make one produce children without a head.*” Woman, IDI – Koro, Northern Uganda

While the main barriers to the uptake of modern family planning methods in Northern Uganda were linked to strong male-partner opposition and fears of possible side effects, in Burundi concerns about male-partner opposition were less common.

The main facilitator for utilisation of family planning services was pressure on limited resources (60%), including land on which cultivation is done. This was considered a growing problem in some of the sites as the incidence of land disputes was reported to have sharply increased, especially following the return of displaced populations.

Factors that were raised only by women in Northern Uganda included the perception of women on contraceptives as ‘men’ or ‘without womanhood’, discouraging some from seeking such services; male-partner opposition to spousal uptake of HIV voluntary counselling and testing (VCT) services for fear of being diagnosed with HIV; and fear of undergoing a caesarean section.

#### LHPs and NGOs

Most of the socio-cultural level factors mentioned by the women were also emphasised by the LHPs and NGO respondents. Factors that were only mentioned by the LHPs and NGOs in Northern Uganda included a great respect for and availability of traditional birth attendants (TBAs) to undertake deliveries in some rural areas (40%); and a cultural perception of pregnancy as a normal condition that may discourage some women from seeking ANC and facility delivery services (50%). In some settings, pregnant women who regularly attended ANC sessions were perceived as ‘*not strong enough*’.

“*People think that when you are pregnant it is a normal condition and you do not have to go to the health facility. They feel that when you go there you are a coward*.” NGO-health provider, IDI – Gulu, Northern Uganda

Respondents to some extent associated the great respect for TBAs to the conflict, as skilled birth attendance was almost non-existent for huge segments of the population during conflict, and TBAs were regarded as heroines within some communities.

Other sociocultural factors were the perception among some men that women on contraceptives are stubborn (difficult to control) and sexually promiscuous (25%); a desire to replace family members lost during the war (85%); and the cultural desire for large family size (77%). These factors also accounted for the often mentioned male-partner opposition to contraceptive use by their spouses. The strong position of the Catholic Church against the use of modern contraceptives was reported to be a key barrier (70%) for the uptake of family services in both Burundi and Northern Uganda, as more than 60% of the population are Catholics. The strong negative impact of the Catholic Church on the uptake of modern family planning services observed among these categories of respondents was *not* mentioned as a major concern among the women respondents.

In Burundi, a few respondents (26%) identified the cultural practice of concealing a pregnancy for the first trimester as a major barrier to early ANC service uptake. This is a practice that is not only limited to uneducated women in rural areas, but also common among educated women in the cities.

The occasional financial costs incurred by women at the level of the facility also discouraged some women from seeking services, while the improved security situation has been an important pull factor.

### Political and health system level

#### Women

Most of the women (95%) in both Burundi and Northern Uganda felt that the most important political and health system level pull factor for uptake of MSRHS is the universal and selective healthcare policy for Uganda and Burundi respectively that facilitates access to services through the removal of user fees. All respondents in Burundi were generally more appreciative of the health system, especially the manner in which they are received and treated at the level of the health facility, compared to their counterparts from Northern Uganda. As such, most respondents from Burundi felt that no barriers existed at the level of the political and health system domain.

“*Women are well treated and whenever you go* [to the health facility] *when you are pregnant, they receive you and they treat you well*.” Woman, IDI – Ruhororo, Burundi

“W*e know that there are nurses at the health centres and hospitals who are ready on a daily basis to receive a woman who is coming to bear a child. They are always ready to help that woman. We thank the government for this. They do not discriminate in receiving patients.*” Woman, FGD – Kinama, Burundi

On the other hand, over half of the women respondents from Northern Uganda felt that although the cost of basic health care is free, some health providers tend to extort money from them. A number of women narrated incidents at the health facility where health providers requested unauthorised financial tips following the delivery of a service.

“*Sometimes you can go* [to the health facility] *and you are told by the nurses to give them some money for the help they have given to you …*” Woman, IDI – Bobi, Northern Uganda

“*When I went to give birth, the nurse told me that ‘since you have given birth well I want you to give me something but don’t tell the in-charge* (supervisor)’. *Then I removed 5,000 Shillings and gave her*.” Woman, IDI – Bungatira, Northern Uganda

Furthermore, the provision of some services such as family planning, ANC, and VCT through mobile outreach clinics and village health teams in the case of Northern Uganda, and TBAs and community health workers in the case of Burundi was also a strong pull factor for the demand for these MSRHS. Of all the women respondents, especially in Northern Uganda, 40% reported that they are drawn to attending ANC services and undertake delivery at a health facility because of material incentives provided along with the services, such as bed nets and delivery kits.

“*Some women go to the health facilities because another woman has gotten that incentive and you hear them saying that ‘if my friend has gotten this there, then I have to also give birth from the hospital in order to get mine’*.” Woman, FGD – Koro, Northern Uganda

A common barrier discouraging some women from seeking facility services was that the attitude of some health providers was occasionally perceived as abusive and degrading to the clients (57%), at times because of their perceived state of poverty. This perceived barrier was, however, very uncommon in Burundi.

“*Some women fear those nurses because they like harassing women when they go to seek for services and some can even abuse you*” Woman, IDI – Bobi, Northern Uganda

Specifically in Burundi, most women (90%) felt that the construction of more health facilities, hence reducing the travel distance, and the recruitment of more health personnel were other facilitators, especially in rural areas. In Northern Uganda, the common barriers raised were the irregular presence and frequent absence of personnel at some facilities (60%), especially in the rural areas, and the policy of insisting that pregnant women must be accompanied by the male partner during some ANC consultations if they are to receive prompt service delivery (63%).

“*If the child the woman is carrying does not have a father, it discourages the woman from going for ANC visits because some facilities require you to come with your husband.*” Woman, IDI – Bungatira, Northern Uganda

A number of women felt that tying prompt ANC service delivery to being accompanied by the male partner unfairly treated women without partners, and women whose partners refused to accompany them or were unavailable for other reasons. The prevailing practice of insisting on male partner involvement was also associated with the reluctance of some women to seek other MSRHS, such as family planning and VCT. In many situations women that were unaccompanied by their spouse were reportedly attended to much later, or even sent away unattended. This practice of prioritizing accompanied women, or even not providing some services to unaccompanied women, was a major concern among some women in Northern Uganda.

“*I would think the health personnel should improve the way they treat mothers when they go for maternal and other services available in the health unit. Not that if they do not go with their husband they should leave without services because there are men who are also very difficult to deal with and so their wives should not be dropped out from services because of their husband’s conduct.*” Woman, IDI – Bobi, Northern Uganda

#### LHPs and NGOs

The political and health system level factors that were identified by the health providers and NGOs were highly similar to those reported by the women. The common facilitating factors that emerged across the study sites included the policy of removal of user-fees (100%), the increasing level of community sensitization on health issues (90%), the prohibition of TBAs from undertaking deliveries, which had directly pushed some women to deliver at health facilities (75%), and the delivery of some services at community level.

In Burundi, the introduction of the performance-based financing (PBF) programme was highlighted as the most important facilitating factor to the delivery and uptake of MSRHS (100%). Through the PBF scheme health personnel are remunerated specifically for the quantity and quality of specific services provided in addition to their regular salary. Facilities are also better stocked with basic supplies than before, the range of services offered has increased, and more lay health workers have been trained from the community to intensify community health sensitization activities. Also, competent personnel tend to always be at the facility, TBAs have been trained and assigned a new role in health promotion and community sensitisation, and the attitude of personnel towards the clients has reportedly improved. All these have encouraged more women to seek MSRHS. On the downside, some respondents (25%) felt that the strong increase in the number of women seeking MSRHS following the introduction of the selective health care and PBF policies has not been sufficiently matched with a corresponding increase in the number of skilled personnel at the facility, nor in the quantity of medical supplies. The end result has been a decline in service quality and delays in the provision of services, which has negatively affected the demand for some services.

Some facilitating factors that were mentioned only by LHPs and NGOs in Northern Uganda are effectiveness in the integration and follow-up of clients, especially in the domains of VCT and prevention of mother-to-child transmission of HIV; professional competence of personnel with respect to safeguarding clients’ privacy and confidentiality; payment of the cost for skilled birth attendance and related services at a reputable private hospital by some local politicians; and availability of youth-focused and youth-friendly services. Moreover, the availability of free antiviral therapy coupled with the provision of nutrition support for HIV positive mothers, and the provision of some incentives (such as a delivery kit and a washing basin) for women who deliver at the facility, were also important pull factors. The main barriers mentioned only by LHPs and NGOs were poor management of pregnant teenagers and teenage mothers; the poor drug supply policy and regular stock-out of some essential supplies at the facility level; and in some areas, the poor coordination among NGOs, health facilities and the district health office affecting the pattern of service delivery.

## Discussion

This study has demonstrated that a complex and inter-related set of factors affect women’s utilisation of MSRHS in post-conflict settings, and that armed conflict are among them. These factors cut across the individual, socio-cultural, and political and health system spheres, and the main determinants include women’s fear of developing pregnancy-related complications, situation of women empowerment and support at the community and household levels, removal of user-fees, proximity to the health facility, and attitudes of health providers. The main negative effects on family planning service uptake related to the exposure to conflict were associated with a generally low level of appreciation of the importance of some services, due to low educational attainment partly as a result of the conflict. Another effect has been a strong cultural desire for a large family size, especially among men, partly as a response to the loss of family members during the conflict. Furthermore, the disruption of infrastructural development such as roads and health facilities during the conflict, means that proximity to functional health facilities for many rural dwellers remains a considerable problem in some areas. While related studies have been undertaken in Uganda, largely employing a quantitative design, we are not aware of any such studies undertaken in Burundi.

Our findings are consistent with those of other researchers in related settings. Previous studies in Northern Uganda have identified lack of finance, of information, and of decision-making powers as key challenges to access to health care services for women [30]. Also, the abusive and unwelcoming attitude of some health providers towards women, financial demands by some health providers, and uncooperative husbands, have been reported in other regions of Uganda as important barriers to the uptake of family planning, ANC visits, and other health services by women [31]. A systematic review of access to and utilisation of health services for the poor in Uganda [32] identified distance to service points, perceived quality of care, and availability of drugs as key determinants. In addition the review concluded that perceived lack of skilled staff in public facilities, late referrals, health worker attitudes, costs of care, and lack of knowledge were important barriers to service utilisation. Although many women appreciate the importance of ANC visits and facility delivery, when they cannot find someone to take care of their families, (especially their children) while they are away at the facility, they opt not to go, as was observed in post-conflict Sierra Leone [33]. In post-conflict Timor-Leste, women’s choice of delivery in a health facility has been linked to previous perinatal deaths or complications, such as prolonged or painful labour, bleeding, or referral in a past pregnancy, as well as the parity status, with primiparous women more likely to deliver at the facility [29]. In post-conflict Liberia, Lori et al. [34] reported that there was a strong sense of secrecy around pregnancy and childbirth, similar to our observation in Burundi, and distrust of the health care system among a proportion of the population, factors that in our study were associated with late attendance of ANC consultations and possibly with home deliveries among some women. Secrecy around such issues might be linked to concerns about witchcraft, in particular that an enemy may bewitch the unborn child or prolong its delivery. Similar views were expressed by some of our study participants. During the 2006 conflict in Lebanon, Kabakian-Khasholian et al. [35] equally observed that the key determinants for seeking maternal care were the availability of health services and experiences of complications. In some conflict settings, the choice of place of delivery is affected by the availability of appropriate clothing to wear to the facility, and the preference of key decision makers in the family, such as mothers-in-law and husbands [36].

In the aftermath of the internal conflict in Timor-Leste in 2006, the country was plagued with similar challenges to those we observed in Burundi and Northern Uganda, and one key response employed by the authorities was the institution of a maternity waiting camp for pregnant women [37]. At one of the facilities we visited in Northern Uganda, such a home was recently introduced especially to deal with pre-identified clients in rural remote areas with the risk of an abnormal delivery. Although this practice seems to be uncommon in our study settings it might be an important intervention to extend to other major health facilities. Accommodating the women and their companions may be a particularly important intervention for those who have to travel over a long distance to come to the facility.

Both Uganda and Burundi have waived user fees for maternal health related services; Uganda introduced a universal healthcare policy in March 2001, while Burundi introduced a selective healthcare policy for women giving birth and children under 5 years in May 2006. This policy seems to be the most important determinant of women’s uptake of MSRHS in our study settings, highlighting the importance of financial barriers in determining the demand for health services. A study in rural Burkina Faso showed that substantial reductions in user fees for ANC and skilled attendance at birth improved equity in access to these services across socio-economic groups, but did not ensure that all women benefited from the services [38]. These observations highlight the importance of also focusing on policies aimed at addressing other barriers. For instance, the current strategy of community provision of some MSRHS such as contraceptives, ANC, and postnatal care through mobile outreaches and local community structures, including traditional birth attendants, community health workers and village health teams, is a welcome model for delivering services, and needs to be strengthened. Furthermore, the level of engagement of the health system and other key community structures with males in the community on the importance of utilisation of MSRHS, including contraceptive uptake, also has to be intensified. Men might not have been appropriately engaged on these issues, and their knowledge of the services may be erroneous, which possibly accounts for the level of resistance that has been observed among some men vis-à-vis the uptake of MSRHS. Health providers might therefore have to coin their messages more efficiently to enhance male partner support for the utilisation of maternal and child health services. For example, a study of Northern Uganda concluded that the introduction of community and health facility capacity strengthening interventions such as training of health workers, provision of medical supplies including delivery kits, and community mobilization using village health teams, dance, drama and “male partner access clubs”, led to improvements in first ANC visit attendance, in VCT service uptake for attendants of first ANC visits, in facility delivery, and in VCT service uptake by couples [39]. While the current free healthcare policy for pregnant women and children under five has had a positive influence on the number of women going for ANC and facility delivery, other associated expenses such as transportation to the health facility, food to eat, clothes for the baby and the mother, and care for the other children at home when the mother is away continue to prevent some women from utilising ANC and facility-based delivery services. Similar observations in Timor-Leste are reported by Wild et al. [29]. In war-torn Afghanistan, Hadi et al. found that with appropriate conditions in place, many women and families will continue to seek facility-based delivery [40]. These conditions include providing free services and transport facilities at night, incentives to health providers, maintaining privacy in the delivery room, and the quality of services.

In many settings where stimulating demand for health services has largely been sought through the removal of user fees, but where proper planning and coordination has been lacking, other challenges on the supply side have arisen [41,42]. This happened in Burundi in May 2006 following the sudden abolition by the president of all user fees for children under five, and for women giving birth in all public health centres and hospitals. This was closely followed by a reduction in financial flows to the facilities, resulting in frequent drug stock-outs, reduced quality of the services, and disruption of the referral system [42]. These are similar challenges to those that we observed across the sites, although these challenges were more acute in the case of Northern Uganda. In Burundi, the nationwide introduction of the PBF programme in April 2010 to complement the earlier introduced free health care policy for children under five and pregnant women, seems to have mitigated some of the challenges that were observed following the introduction of the free healthcare policy. This has led to a generally more positive perception of the health system among women in Burundi compared to the women in Northern Uganda, as we observed in our study. The PBF scheme is a supply-side results-based financing programme which involves a ‘fee-for-service–conditional-on-quality of care’ mechanism that rewards hospitals and health facilities with monthly payments determined by service utilisation levels and performance on quality measures [43]. In the absence of a similar and well-coordinated personnel remuneration system like the PBF, health personnel in Northern Uganda may be more demoralized, less enthusiastic in the delivery of basic health services, and more prone to request unofficial payments from clients. The initial challenges faced by Burundi in the wake of the introduction of the selective healthcare policy, and nowadays in Northern Uganda, where a universal healthcare policy is in place, points to the importance of careful planning, implementation and coordination of such policies. However, failure to do so may seriously compromise the quality of services, as was observed across the study sites, and especially those in Northern Uganda. While the positive impact of the PBF programme on the utilisation and quality of maternal and child health services was widely reported by participants in Burundi, a few participants equally acknowledged that challenges with respect to staff burn-out and service quality as a result of the increasing demand for services remain. Although a number of post-conflict countries in Africa including Burundi and Rwanda have rolled-out nationwide PBF schemes as a means of improving health worker performance and as a tool for health sector reform, Ireland et al. have questioned the validity of PBF as a tool for health sector reform. They argue that the “debate surrounding PBF is biased by insufficient and unsubstantiated evidence that does not adequately take account of context nor disentangle the various elements of the PBF package” [44] (p. 695).

Based on our findings and those of previous studies [12-16], the determinants seem to be largely the same in post-conflict and non-conflict settings except for the fact that *the barriers in post-conflict settings tend to be more widespread and exacerbated*. We demonstrate that exposure to armed conflict affected women’s utilisation of MSRHS mainly through low educational attainment for both men and women translating into ignorance of the importance of health services and into high levels of impoverishment. Another commonly observed effect was the strong desire, especially among men, to replace lost family members, resulting in their general opposition to modern contraceptives. Working in the opposite direction, great pressure on limited land for cultivation coupled with reported increased incidence of land disputes as conflict-displaced populations return to their communities, appear to encourage some women to consider modern family planning services for birth control.

Our findings also highlight some similarities and differences in the perceived determinants of women’s uptake of MSRHS between and within the categories of participants and study settings. For example, almost all factors identified by the women were also highlighted by the LHP and NGO respondents. This is not particularly surprising, as the latter serve within the communities where these women reside, and have a generally good knowledge of the socio-cultural context of these women. Also, a number of the NGOs and local health providers have local community projects within our study areas that may further improve their level of engagement with the women in those communities. This possible practice of engagement of health personnel with local communities is worth encouraging and supporting as it may improve the delivery of services, thus providing better client satisfaction. However, while the LHP and NGO respondents across the study sites perceived the Catholic Church as having a very strong negative effect on the uptake of modern contraceptives, this was not a concern among the women respondents. The major barriers for the women were opposition from their male partner and the fear of possible side effects. The non-mention of a strong religious influence on modern contraceptives uptake by the women might reflect the fact that the religious values that some women hold may not necessarily be in keeping with the official teachings of their religion, or that their local cultural values may have a much stronger impact on their belief systems. Alternatively, the women might simply not want to apportion blame on their religion as a sign of respect. It is also important to note that the issue of seeking facility delivery in Burundi was strongly associated with the desire to obtain a birth certificate for the child. This highlights the importance that women in rural Burundi place on the free healthcare policy, as the birth certificate of the child might be required at times in public facilities before services are provided free of charge. The issue of limited land that has served as a facilitator to family planning uptake was raised *only* by the women, across the study sites. This might reflect the reality these women go through on a daily basis to raise their children and put food on the table for their families. Since the women were largely based in rural areas, with farming as their main occupation, they might have personally experienced the challenges of having a large family living off a limited piece of land, and how such pressure affects household- and community cohesion. This may explain why some women disregard personal risk and seek for concealable modern contraceptives against the backdrop of male-partner- and cultural opposition. The other concerns raised about the uptake of modern contraceptives are not unique to our study. A study in Ghana found that a third of women considered modern contraceptives as unsafe, 20% reported opposition from their male partner as a barrier to uptake, and 65% of users reported at least one side-effect [45]. Therefore, in order to improve the uptake and continual usage of modern contraceptives in these areas, these concerns have to be addressed.

The challenges of delivering health care and rebuilding health systems in conflict and post-conflict settings have been well acknowledged. The major challenges are the lack of security; acute shortage of skilled health professionals due to migration to safer areas; lack of infrastructure and medical supplies and drugs; obstruction of access to health facilities by warring parties; security forces harassing, arresting and prosecuting health providers; poor coordination among government, health care providers and humanitarian organizations; and assaults on patients within hospitals, among others [46-50]. These challenges make the health system non-functional, resulting in limited availability of, limited access to, and poor quality of health services. As such, rebuilding health systems must take into consideration the prevailing challenges to ensure efficient use of limited resources and provide maximum impact. In this regard, experts have recommended that health system strengthening programmes in such settings should put more emphasis in the short-term on the provision of primary health care services, using existing human resources for health, community structures, NGOs and mobile outreach clinics [51]. Programmes such as the renovation and construction of health facilities and the development of human resources for healthcare are more likely to succeed in the medium- and long term. This happens to be the approach that both governments have eventually embarked on, although in the earlier post-conflict years in Northern Uganda so many resources were channeled into the construction of health facilities, especially in rural areas, that to date many remain non-functional due to acute shortage of human and material resources. A more stepwise approach, rather than thinning out the limited resources over a large area without much progress taking place, could have been more effective. Furthermore, governments of post-conflict settings along with their development partners must carefully design the core elements of the health system to provide reliable essential health while ensuring that it addresses issues around equity, government accountability to citizens, and governments’ capacity to manage important social programs [47].

### Limitations

A limitation of the study was that the women participants were mainly staying within the catchment areas of some local health centre or had regular weekly access to basic healthcare services through mobile outreach clinics. We were unable to recruit women participants in much disadvantaged remote areas that were not regularly served with basic health services. As such, the perspectives of that group of women are not well captured in our study.

## Conclusions

In post-conflict settings, a vast and complex set of factors affect women’s utilisation of MSRHS ranging from the individual, socio-cultural, political to health system levels. The main determinants include the removal of financial barriers to access; level of household, community and facility support for women; proximity to health services; and community perceptions of some services. Exposure to conflict generally exacerbated the barriers to women’s uptake of services, mainly through low educational attainment and stronger cultural desire for increased fertility to replace family members lost to the conflict. To improve women’s uptake of MSRHS in such settings, robust health system strengthening programmes addressing the barriers across the individual, socio-cultural and political spheres are needed. While addressing financial barriers to access is important, attention should also be paid to non-financial barriers. The goal should be developing an equitable and sustainable health system.

## Additional files

Additional file 1:
**Methods.** This is a detailed description of the materials and methods used for undertaking the study. It includes a description of the study settings and participants, data collection, management and analysis methods, collaborative partnership, recruitment of participants and ethical considerations. Additional file 2:
**Data Collection Tool: Interview and Focus Group Discussion Guides.** This is a detailed description of the interview and focus group discussion guides for the various categories of research participants. The guides are for the entire study from which this paper is one of the outcomes. Additional file 3: Table S2. Factors affecting women’s utilisation of Maternal, Sexual and Reproductive Health Services (MSRHS) in post-conflict Burundi and Northern Uganda. This is a summary of the factors affecting women’s utilisation of maternal, sexual and reproductive health services in Burundi and Northern Uganda as perceived by women of reproductive age, local health providers and staff of NGOs working in the domain of maternal and reproductive health. The factors are further classified into individual level factors, socio-cultural level factors and political and health system level factors.
