# Supplementary material for: A qualitative study exploring the determinants of maternal health service uptake in post-conflict Burundi and Northern Uganda
Source: BMC Pregnancy Childbirth. 2015 Feb 5;15:18. doi: 10.1186/s12884-015-0449-8 (PMC4327793; doi:10.1186/s12884-015-0449-8)
Supplement: Additional file 1: — Methods. This is a detailed description of the materials and methods used for undertaking the study. It includes a description of the study settings and participants, data collection, management and analysis methods, collaborative partnership, recruitment of participants and ethical considerations. [file 12884_2015_449_MOESM1_ESM.docx]

**Additional file 1: Methods**

**Introduction**

This paper is part of a two-country qualitative fieldwork looking at the impact of armed conflict on maternal, sexual and reproductive health (MSRH) in some post-conflict settings in sub-Saharan Africa.

**Study settings**

The study was undertaken in two provinces in Burundi (Bujumbura Marie and Ngozi) and a district in Northern Uganda (Gulu). As of 2008 the populations of the provinces of Bujumbura Marie and Ngozi were projected at 411, 385 and 761569 respectively, while the national population was just over 8 million^[[1]](#footnote-1)^. However, the 2013 population of the country was projected at 10.16 million^[[2]](#footnote-2)^. In Burundi participants were recruited from the cities of Bujumbura and Ngozi and the rural and semi-urban communes of Ruhororo in Ngozi Province and Kinama in Bujumbura Marie province respectively, while in Gulu district in Uganda, our participants were recruited from the rural sub-counties of Koro, Bobi and Bungatira, along with Gulu municipality (made up of four sub-counties). The Gulu district is made up of 3 counties, 16 subcounties, 70 parishes and 279 villages, with a population of 374,700^[[3]](#footnote-3)^.

**Map of Burundi (showing Bujumbura and Ngozi provinces)**


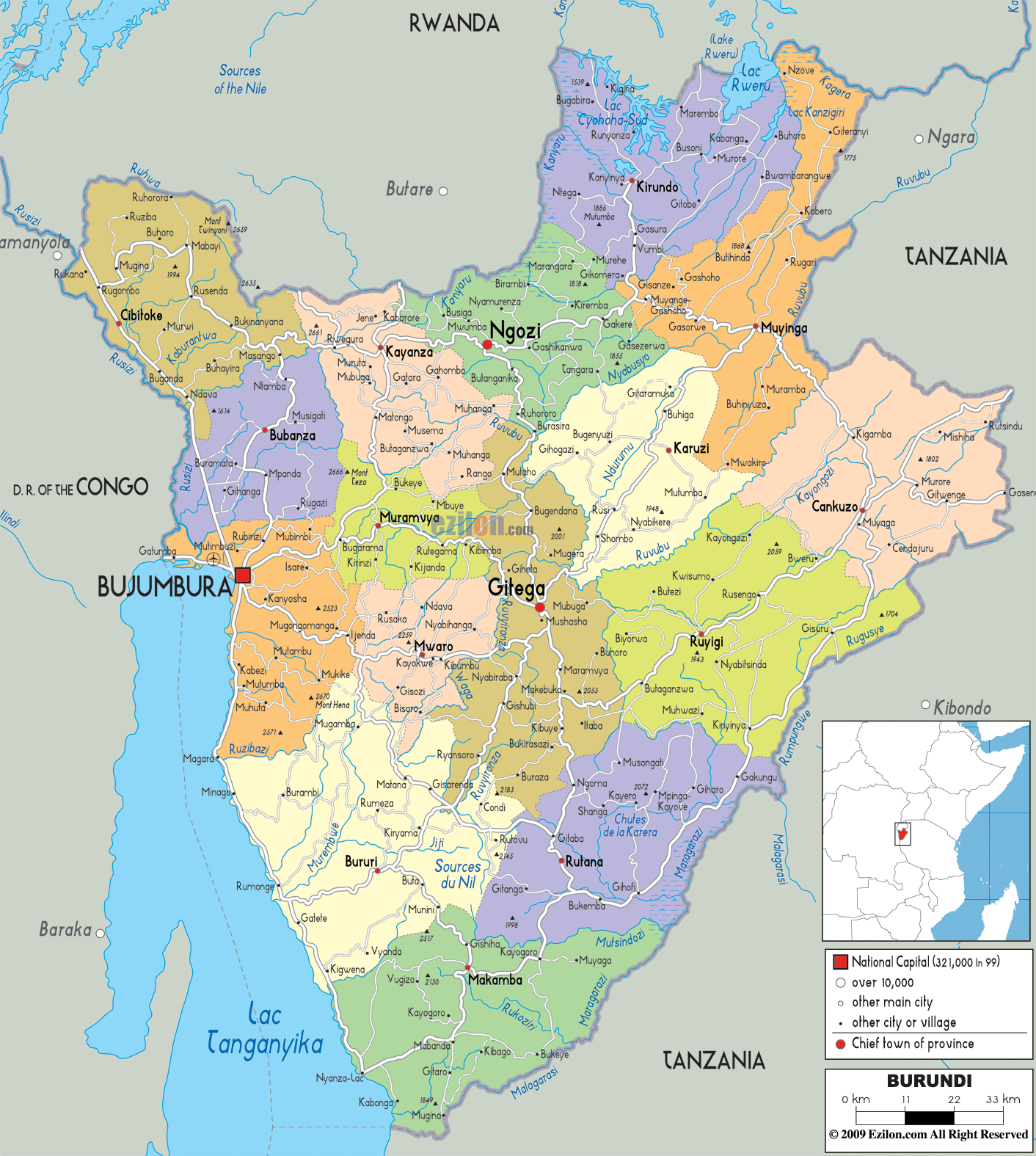


Ngozi & Bujumbura Marie Provinces

Source: Political Map of Burundi (<http://www.ezilon.com/maps/africa/burundi-maps.html>)

**Map of Uganda showing the administrative units (districts) and map of Gulu District**


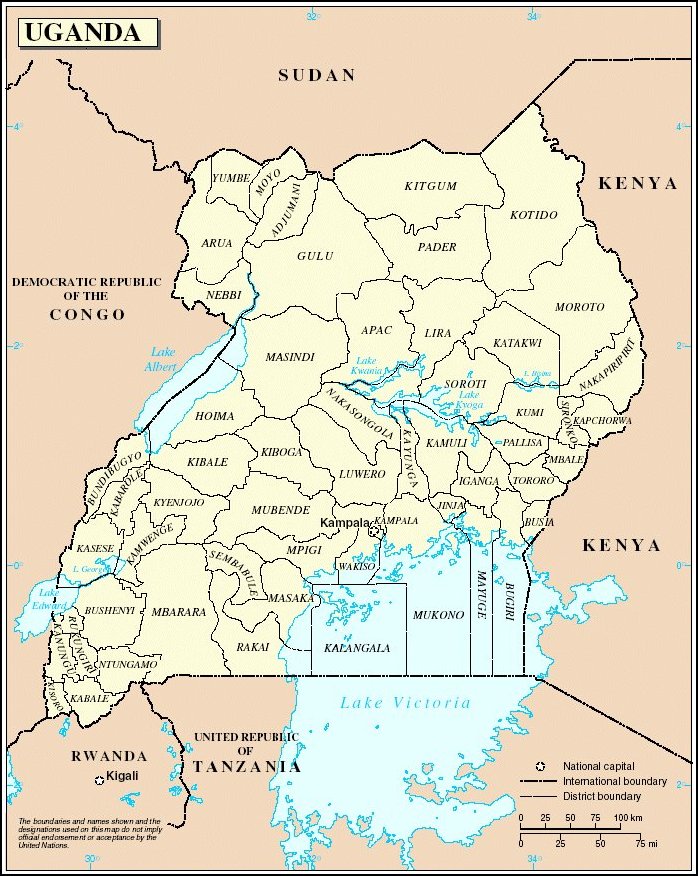

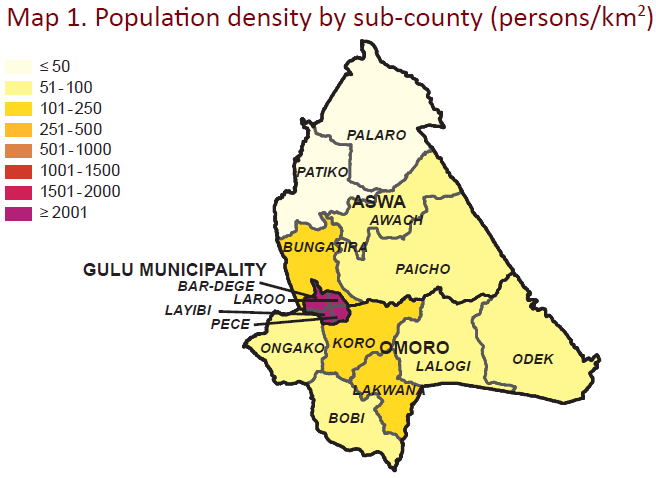


Source: United Nations Map of Uganda by Districts (<http://www.ugandamission.net/aboutug/map1.html>) and Map of Gulu (Ministry of Water & Environment)

**Study Participants**

Study participants were recruited from among non-governmental organizations (NGOs), local health providers and women. The NGOs included local, national and international organizations working in the domain of MSRH, be it at the level of policy support/ technical assistance, health system support and strengthening, delivery of health services, etc. The international NGOs included UN- and the non-UN-based organizations. The local health providers were drawn from clinics, health centres and hospitals and included nurses, midwives and doctors working on MSRH issues in their institutions, mainly in the maternity, antenatal care, and obstetric and gynaecological units. Others also included senior administrators at ministries of health at the provincial, regional or district levels. The women were mainly in their reproductive age and settled in rural or semi-urban areas. In this regard, all interviews and FGDs with women were undertaken only in the communes of Rohororo and Kinama in Burundi, and in the sub-counties of Koro, Bobi and Bungatira in Northern Uganda. Since we were interested in also capturing the effect the conflict had on MSRHS, the NGOs and health providers invited to participate in the study had all developed, supported and/or provided MSRHS during the conflict or shortly after the conflict, while the women had sought or attempted to seek such services during such periods as well.

**Data collection method**

This is an exploratory qualitative study that used in-depth interviews (IDIs) and focus group discussions (FGDs) for data collection. Interviews and FGDs were conducted in the local languages, French or English (where applicable), by the principal investigator (PCC) or by trained local research assistants. A total of 63 IDIs and 8 FGDs involving 115 participants were conducted. The fieldwork took place from June – September 2013. During the interviews and FGDs, field notes were taken. These mainly captured observations such as the physical setting, the human and social environment, informal interactions and unplanned activities, nonverbal communication, formal interactions, and other information of interest. The field notes were used to better appreciate the context in which the interviews and FGDs were undertaken.

**Issues discussed**

The interviews and FGDs focused specifically on the general state of MSRH in Burundi and Northern Uganda, aimed at describing the general state of maternal health and understanding the factors affecting women’s utilization of basic MSRHS, taking into consideration the possible effects of the recent conflict. The detailed guides for the interviews and FGDs for each of the participant categories can be found in Additional file 2. Box 1 shows a sample of some of the questions.

**Box 1: Sample of questions posed to participants during IDIs and FGDs**

1. What factors do you think affects women’s utilisation of health services during pregnancy and childbirth? (explore possible factors such as quality of care/treatment provided by health provider, costs for services, travel distance, lack of knowledge on when to seek care etc ).

2. Have these factors changed over time? (probe to inquire how?).

3. Do you have any ideas how the past conflict might have affected this? (probe to inquire how was the use before and after etc).

**Collaborative partnership**

Prior to arrival in the study area, we undertook an internet search of potential NGOs working in the domain of MSRH in the area. We also approached prominent organizations/institutions working in the domain of MSRH in the area for a list of existing organizations/ institutions working on the ground in that domain. In Burundi, our local partners were UNFPA-Burundi and the Burundi University Research Centre for Economic and Social Development (CURDES) while in Uganda we worked closely with Reproductive Health Uganda (RHU). These partner organizations are involved in health systems strengthening and support in the various settings and will serve as veritable platforms for dissemination of study findings. In each of the settings, research assistants (RAs) were locally recruited.

**Recruitment**

Purposive sampling was the main sampling method employed. For the women, we mainly approached those who had had some experience of the conflict or the period immediately after the conflict. We were particularly interested in those who had given birth during any of those periods in order to capture the challenges they experienced in order to seek health care. For the NGOs and local health personnel, we were also keen to recruit those working in the domain of MSRH and whose interventions/activities are designed with the conflict or post-conflict challenges in mind. The NGO and local health provider personnel were recruited specifically for the role as health professionals involved in the support and delivery of health services to women. As such, their responses were based on what factors they considered affected women’s uptake of the MSRHS they provided or supported.

Upon arrival at a local health institution (hospital, health centre or clinic) and NGO office with a letter of support from the local administrative and/or health office, permission was further obtained from the institutional head to approach an appropriate staff to participate in the study. Most often, the institutional heads choose the most appropriate staff to approach based on the criteria we provided. At health institutions, our participants were mainly drawn from the maternity, antenatal care, and obstetric and gynecological units while at NGOs our participants were mainly maternal and/or reproductive health programme/ project coordinators/ advisors.

Recruitment of women participants was mainly within communities in rural and semi-urban areas. Upon arrival in each community, the research team presented themselves to the local council office or community head, usually with a letter(s) of support from the district or provincial health office and/or local collaborating partner. The council or community then approved our entrance into the community, where we could approach local households and invite local women to participant in our study. Participants were informed about the study goal and procedure in the local language and were requested to ask questions for clarity. Only those that provided their consent following the information session participated in the study. At any point in time, we made an effort to clarify that we were independent researchers and not an NGO and as such were not involved in any way to provision of MSRHS in the area.

**Conducting interviews and FGDs**

All individual interviews and FGDs with women were held within the community, mainly in their homes or nearby in some community space. Interviews and FGDs for NGO staff and local health personnel were held mainly at their places of work, and lawn of a local hotel. All interviews in French and the local languages were undertaken by the trained local RAs while all the English interviews were undertaken by the principal investigator (PCC). Soft drinks, tea or coffee was provided to FGD Participants during the discussion. We also provided transport reimbursement to FGD participants.

**Research Assistants and guides**

In each of the settings, RAs and guides were recruited locally. A total of 6 RAs and guides were recruited and trained across the study sites. All RAs understood the local language(s) plus English and/or French and were educated up to the university level. In Burundi interviews and FGDs were mainly held in the French or Kirundi language, while in Northern Uganda they were held in the English or Luo language.

**Data Management and Analysis**

All interviews and FGDs were audio-recorded and later transcribed and translated into English (where applicable). The English transcripts were the imported into the QRS Nvivo (QSR International, 2012). Considering the multidisciplinary nature of the research team and given that the data are mainly made up of semi-structured interview transcripts, the framework method [28] was used to manage and analyze the data. Three team members open-coded the transcripts on Nvivo and Microsoft® Word (where the texts of interest are highlight and the code first labeled using the ‘*New Commen*t’ sub-menu under the ‘*Review*’ menu). Microsoft® Word was used for coding and analysis by one of the co-authors who did not have access to Nvivo. The codes were descriptions or labels of specific ideas as the transcripts were read. Two team members reviewed the codes that were developed and the inter-coder reliability was high. Inter-related or similar codes were then clustered into different categories, and the categories were subsequently grouped into specific themes. The themes were inductively and deductively developed. Inductive means that they were anticipated from the design of the interview and FGD guides and consciously explored in the interviews and FGDs. Deductive means that they were not anticipated during the design and identified during the review of the transcripts. There was therefore a constant interplay between data collection, analysis and theme development, with new and dominant ideas that emerged in earlier interviews and FGDs being explored deeper in subsequent and later interviews and discussions. The themes were also developed taking into consideration the main factors affecting women’s utilization of maternal health services proposed by Wild et al.’s [29] multilayered explanatory model. According to the model, the utilization of maternal health services is affected by different layers of influences ranging from individual, social, cultural, political to health system spheres. The theme development was jointly undertaken by three team members.

**Ethics and administrative approval**s

Ethics approval for the study was obtained from the Regional Committee for Medical and Health Research Ethics, South-East (Norway); le Comité National d’Ethique pour la Protection des êtres Humains Participant à la Recherche Biomédicale et Comportementale (Burundi); and Gulu University Institutional Review Committee (Uganda). We also received permission from local administrative and health authorities. All participants/informants gave their informed consent before participating in the study and their anonymity, privacy and confidentiality was respected. Written or oral consent were appropriate and acceptable for our settings and approved by the relevant ethics committees. All oral consents were audio-recorded.

1. Projet Appui aux Programme de la Population, APP. Burundi: Population Statistics. <http://www.humanitarianresponse.info/operations/burundi/dataset/burundi-population-statistics> [↑](#footnote-ref-1)
2. Population Division, UN Department of Economic and Social Affairs. World Population 2012. <http://www.un.org/en/development/desa/population/publications/pdf/trends/WPP2012_Wallchart.pdf> [↑](#footnote-ref-2)
3. Directorate of Water Development, Ministry of Water & Environment, Uganda, 2010. Gulu. <http://www.mwe.go.ug/index.php?option=com_docman&task=doc_download&gid=38&Itemid=223> [↑](#footnote-ref-3)
