# Supplementary material for: A qualitative study exploring the determinants of maternal health service uptake in post-conflict Burundi and Northern Uganda
Source: BMC Pregnancy Childbirth. 2015 Feb 5;15:18. doi: 10.1186/s12884-015-0449-8 (PMC4327793; doi:10.1186/s12884-015-0449-8)
Supplement: Additional file 2: — Data Collection Tool: Interview and Focus Group Discussion Guides. This is a detailed description of the interview and focus group discussion guides for the various categories of research participants. The guides are for the entire study from which this paper is one of the outcomes. [file 12884_2015_449_MOESM2_ESM.doc]

**Additional file 2:** **Data Collection Tool: Interview and Focus Group Discussion Guides**

**Interview/FGD guide for Qualitative study**

Key issues explored included:

1. Knowledge of obstetric danger signs among pregnant women;
2. The role of traditional birth attendants in early recognition of the danger signs and timely referral of women;
3. The state of the communication and transport network to referral health facilities;
4. The existence of community finance and transport schemes to ease referral of women to health facilities;
5. The existence of referral facilities and adequacy of health personnel at the facility
6. The state of existing Emergency Obstetric Care (EmOC) services (present/absent; basic/comprehensive, etc);
7. The availability, utilization and quality of EmOC (including accessibility and opening hours) and other MSRH services prior, during, and after the conflict.
8. The main causes of maternal deaths (haemorrhage, infection, hypertensive disorders, abortion complications and obstructed labour), classified into the three-delays model (Thaddeus & Maine, 1994), and if the causes were managed in line with the proposed model interventions.
9. Descriptive accounts of the effects of the conflict on the accessibility, affordability and quality of MSRH services in their specific area/region.
10. Eyewitness accounts of specific negative consequences of the conflict on MSRH
11. Strategies put in place to ameliorate the prevailing situation/coping strategies.

**Interview guide: Women of Reproductive Age and women who lived during the conflict era**

Introductory questions (creating rapport): Mainly demographic info. (area of residence, migration status, exposure to conflict (direct/indirect), occupation, age, educational level, number of children etc. )

**Pregnancy danger signs, complications and utilization of healthcare services**

- There are some signs during pregnancy and childbirth that indicates that the health of the woman and/or baby may be in danger. Do you know about these signs?
- Can you talk to me about some of these signs? (probe if she could describe any of the signs below: Fitting; Swollen feet, hands and/or face; Severe headache; bleeding; High fever after childbirth; Labour lasting more than 12 hours; Hand, foot or cord comes first; Placenta still has not come out after 30 minutes)
- How can you recognize these signs when they occur? How can you know that a pregnant woman’s health and wellbeing is in danger?
- When & Where did you learn about these pregnancy danger signs? (probe about these options: health facility; radio; community meetings; community health workers etc )
- How was your knowledge about these signs before the conflict?
- How has the conflict affected your knowledge about these danger signs? (probe if it has improved or not and how).
- During and after the conflict, did you experience any of these signs when you were pregnant?:
- Fitting;
- Swollen feet, hands and/or face;
- headache;
- bleeding;
- High fever after childbirth;
- Labour lasting more than 12 hours;
- Hand, foot or cord comes first;
- Placenta still has not come out after 30 minutes
- Did you disclose these signs to your care giver or anyone else
- What did you/ your ‘carer’ do when you experienced this?
- Were you ever pregnant during and immediately after the conflict?
- Where did you deliver your baby?
- How was your delivery? (probe whether she delivered well without any problems etc)
- Did you experience any complications? (probe more about the nature of the complication). If yes, were you told the cause of the complication?
- Around this area, what types of complications do women have in late pregnancy, labour, delivery and the period immediately after birth?
- How was the situation before the conflict?
- In your opinion how has the past conflict affected this?
- In the past, when women experienced serious complications during pregnancy, delivery or after birth, what did the community do? (probe if there are any community insurance schemes, assistance in transporting the woman to a health facility, contributing funds etc).
- How has this evolved over time, taking into consideration the conflict that happened here? (probe whether the conflict has engendered more community cohesion, participation in joint community activities, created distrust among community members etc)
- In your view are women with complications able to quickly access the care they need?
- Why or why not? (probe for possible difficulties in accessing care such as security concerns, distance, difficulty to access transport, funds, terrain etc).
- How has the conflict affected this?
- What factors do you think affect women’s utilization of health services during pregnancy and childbirth? (explore possible factors such quality of care/treatment provided by health provider, costs for services, travel distance, lack of knowledge on when to seek care etc ).
- Have these factors changed over time? (probe to inquire how?).
- Any ideas how the past conflict might have affected this? (probe to inquire how was the use before and after etc).
- In your view, what would be the most effective way to increase the use of health facilities during pregnancy and childbirth?
- What are the various maternal and reproductive health services provided at your local health facility (probe about family planning, VCT, PMTCT, screening for STIs and reproductive health-related cancers etc)
- What factors in your view encourage women in this area to use maternal, sexual and reproductive health (MSRH) services in general (family planning, VCT, PMTCT, screening for STIs and reproductive health-related cancers etc)?
- What about factors that discourage the use of these services? (probe about user fees, quality of care, etc ).

**Traditional Birth Attendants (TBAs) and community**

- In some parts of the world many women deliver their babies with the assistance of traditional birth attendants (traditional midwives). What is the situation in this region?
- How are they regarded within the community? (probe if they are given more respect and whether many women continue to go to them despite the presence of a health facility nearby etc).
- In addition to assisting in childbirth what other childbirth-related activities do traditional midwives carry out in the community? (probe about their possible role in the early recognition of pregnancy danger signs and timely referral of pregnant women to the health facility etc.)
- How has the role of these traditional midwives evolved over time especially during the conflict? (probe whether because of possible difficulties in accessing health facilities during the conflict, many women and their family depended on them for childbirth **OR** the coming of many organizations/ institutions providing health services as a result of the conflict has reduced the influence/ prominence of traditional midwives **OR** their role has changed over time from delivery in the past towards early recognition of danger signs and timely referral of women to health facilities etc).
- Are there any community finance/insurance schemes and transport schemes to assist with birth-preparedness and transport of pregnant women who need medical attention to the health facility?
- How do they operate?
- How do people become members?
- Is such membership opened to all?
- In your view, are these schemes effective and making a difference in the lives of women in the communities?
- In what way do you think the communities could contribute towards improving MSRH of women in this area?
- At the level of the community, are you aware of any activities/ initiatives aimed at improving the quality of and access to maternal, sexual and reproductive health services following the end of the conflict? (probe to explore issues like emergency loan funds, transport service, health insurance scheme etc).
- Can you kindly describe some of these community initiatives?

**Impact of conflict**

- Can you describe your experience in accessing emergency obstetrics care (EmOC) at your local health facility: (a)before, (b)during and (c)after the conflict?
- How will you describe the quality of the services provided?
- What about the opening hours? Did you find them convenient for somebody like yourself?
- How will you describe the treatment you received at the facility? (probe for quality of care provided etc)
- How have the services evolved over time? (probe whether the services have improved, deteriorated or unchanged, and how)
- Can you kindly describe some eyewitness accounts of the negative consequences of the war on MSRH? (probe about what happened, who was involved and how widespread were issues like those etc)

**Interview guide: Local Health Personnel (Doctors, midwives, nurses, CHWs etc) AND International NGO staff**

**General health status**

- What role do you play in maternal, sexual and reproductive health (MSRH) services in your district?
- How satisfied are you with the availability of these services in your area? Why?
- How satisfied are you with the **overall** quality of services in your area? Why?
- What factors do you think affect women’s utilization of health services during pregnancy and childbirth? (explore possible factors such quality of care/treatment provided by health provider, costs for services, travel distance, knowledge on when to seek care etc ).
- Have these factors changed over time? (probe to inquire how?).
- Any ideas how the past conflict might have affected this? (probe to inquire how was the use before and after etc).
- In your view, what would be the most effective way to increase the use of health facilities during pregnancy and childbirth?

**TBAs**

- In some regions of the world traditional birth attendants (TBAs) have been formally integrated within the health system. What is the situation in this region? (probe if any relationship/link exists between TBAs and the formal health system; how they are regarded within the formal health system; if they jointly undertake some activities with health personnel within the formal health system etc)
- Are there any occasions when the TBAs and health personnel work together? (probe the type and nature of the events; the nature of the working relationship during such events etc.)
- What role do the TBAs play in maternal health in this region and the country at large? (probe if apart of delivery in some settings they also play a role in the recognition and timely referral of women with delivery complications to the health facility)
- Do TBAs refer cases to you? How often? And what clinical stage?

**Communication network**

- How will you describe the state of communication (road network, referral network etc) between primary health facilities and referral health facilities with respect to maternal health issues?
- How is such communication undertaken between these facilities?
- Is there any dedicated communication network between these facilities?
- How effective is such a network?
- How will you describe the state of the existing transport network: (a)before, (b)during and (c)after the conflict from primary health and referral health facilities?

**Impact of war on MSRH**

In the next set of questions we wish to capture some information on how the conflict might have affected MSRH in the country in general and specifically in the areas where fighting took place. If you can also explain with some examples that will be greatly appreciated.

- In many parts of the world, many women and their families suffer from delivery complications because they are not aware about some pregnancy danger signs. What is the situation in this region?
- To what extent could this be associated to the conflict?
- In your opinion, how did the conflict/war affect accessibility to MSRH services?
- What about the impact on affordability of these services?
- And impact on the quality of the services? (probe more for examples and any clarifications).
- Can you kindly describe some eyewitness accounts of the negative consequences of the war on MSRH? (probe about what happened, who was involved and how widespread were issues like those etc )
- As health personnel/ relief workers, what were the key strategies that you people utilized to ameliorate/ lessen the consequences of the war on MSRH?
- What were some of the difficulties that you people encountered and how did you manage to address these difficulties?
- What were the principal causes of maternal deaths during the conflict?
- How did this differ before and after the conflict?
- What strategies were adopted to improve on the situation? (probe some detail description and how the strategies helped to reduce maternal deaths).
- How will you describe the state of EmOC during the war?
- What about their availability for women who needed them?
- What about the rate of utilization/ demand for the services?
- And finally, the quality of the services provided?
- How did these services differ with those before and after the war?
- Around this area, what types of complications do women have in late pregnancy, labour, delivery and the period immediately after birth?
- How was the situation before the conflict?
- In your opinion how has the past conflict affected this?
- Can you kindly recall of any occasion when a woman experienced a complication during pregnancy, childbirth or after delivery? What kind of complication(s) occurred?,
- What steps were taken and by whom to address the problem?
- What was the outcome of the case(s)?
- In your opinion, could the conflict have played a role in the occurrence and management of these complications?
- In the past, when women experienced serious complications during pregnancy, delivery or after birth, what did the community do? (probe if there are any community insurance schemes, assistance in transporting the woman to a health facility, contributing funds etc).
- How has this evolved over time, taking into consideration the conflict that happened here? (probe whether the conflict has engendered more community cohesion, participation in joint community activities, created distrust among community members etc)
- Are women with complications able to quickly access the care they need?
- Why or why not? (probe for possible difficulties in accessing care such as security concerns, distance, difficulty to access transport, funds etc).
- How has the conflict affected this?
- When women with complications reach a health facility, do they get the care they need?
- In your opinion what is the: (a)**quality** and (b) **availability** of care offered to women when they come to the health facility? (probe to know why the quality and availability might not always be the best? ).
- To what extent might the conflict have affected this?
- What factors in your view encourage women in this area to use MSRH services in general (FP, VCT, screening for STIs and reproductive health related cancers etc)?
- What about factors that discourage the use of these services? (probe about user fees, quality of care, etc ).
- In what way do you think the communities could contribute towards improving MSRH of women in this area?
- At the level of the community, are you aware of any activities/initiatives aimed at improving the quality of and access to maternal, sexual and reproductive health services following the end of the conflict? (probe to explore issues like emergency loan funds, transport service, health insurance schemes etc).
- Can you kindly describe some of these community initiatives?

**Causes of maternal deaths**

Generally, maternal deaths have been classified as occurring because of 3 major delays (decision to seek care; identifying and reaching a health facility; and reception of appropriate care and treatment at the facility).

- To what extent are these delays implicated in maternal deaths in this region?
- In your view which of the delays is most implicated in maternal deaths in the region? (probe to know why).
- How have these delays evolved in course of the conflict? (probe to check if the main delay associated with maternal deaths has been the same before, during and after the conflict. For instance decision to seek care might have been very common in the **past** but improved knowledge of danger signs within the communities might have resulted in very few deaths after the conflict associated with such a delay etc).
- What steps are currently undertaken to reduce maternal deaths from each of these delays in this region? (probe about sensitization of community on pregnancy danger signs, provision of transport, development of community insurance and saving schemes for health emergencies, improved road networks, improved referral system etc ).

**Guide for the FGD (for local health personnel and international NGO staff)**

1. Introduction about the study (purpose, procedure, potential risks and anticipated benefits, reporting of findings, funder, informed consent, permission to record etc)
2. Brief introduction of group participants and their roles in maternal, sexual and reproductive health (MSRH) where they work
3. Discussion proper:
4. First set of discussion themes

- Community (including women’s) knowledge of pregnancy danger signs (current status; evolution before, during and after the conflict; role of health system in community education and advocacy on these signs; etc)
- Traditional birth attendants (existence within the communities; nature of services undertaken; integration within the formal health system (?); existing link/relationship with health personnel within the formal health system; possible role in the early recognition of danger signs and referral to health facility; etc)

1. Second set of discussion themes

- Maternal health (current status; trend before, during and after conflict; etc)
- Main causes of maternal deaths (cause-specific mortality; and mortality with respect to the 3-delays model) (current status; trend before, during and after conflict; etc)
- Current health system interventions carried out to address the delays in the region
- Emergency Obstetric Care (EmOC):

1. current status (main signal functions provided, basic EmOC & Comprehensive EmOC facilities etc
2. Trend before, during and after conflict
3. Accessibility of services to women, especially those affected by conflict
4. Availability of services in conflict-affected communities
5. Utilization of services in the region
6. Perception of the quality of services provided
7. Opening hours of facilities
8. Availability of medical supplies
9. Third set of discussion themes

- State of communication network (roads, transport system, referral system, ambulance service etc) between primary health facilities and referral health facilities for maternal, sexual and reproductive health (current status; evolution before, during and after the conflict etc.)
- Community finance, insurance and transport schemes to ease transport of women and/or baby needing medical attention to health facilities (existence(?); coverage; effectiveness; management etc)

1. Fourth set of discussion themes

- Impact of conflict on maternal, sexual and reproductive health (with examples):

1. Individual
2. Health system (personnel, infrastructure, resources etc)
3. Accessibility to health services
4. Affordability
5. Quality
6. Progress towards MDG 5 (specific channel)
7. Resource mobilization

- Eyewitness accounts of specific negative consequences of conflict on MSRH
- Strategies developed to ameliorate the impact of the consequences of the conflict

1. Closing of discussion (thanking the participants; reminding them about commitments for confidentiality and privacy from research team; possibility of re-contacting them for an in-depth interview etc).

**Guide for the FGD (for women)**

Introductory words about the study (purpose, procedure, potential risks and anticipated benefits, reporting of findings, funder, informed consent, permission to record etc)

**Discussion proper**:

First set of discussion themes

**Knowledge of pregnancy danger signs and TBAs**

- Women’s knowledge of pregnancy danger signs (current status/identification of these signs; when they first heard about the signs (before or after their first childbirth); source(s) of information for these signs; community knowledge of these signs; actions taken when such signs happen; evolution before, during and after the conflict; role of health system in community education and advocacy on these signs; etc)
- Traditional birth attendants (existence within the communities; coverage (how common are they); other childbirth-related activities undertaken in the community; possible role in the early recognition of danger signs and referral to health facility; experiences with childbirth using a TBA (perceived quality of service); preference for TBAs over health personnel?; evolution of the role of TBAs vis-à-vis the conflict etc)

Second set of discussion themes

**Transport network to health facilities**

- Current state (nature of roads, access to transportation etc)
- Evolution before, during and after the conflict
- Community initiatives to facilitate transport of women and children needing medical assistance to the health facility
- Existence of insurance scheme, and birth-preparedness schemes within the community; coverage; effectiveness; management etc

Third set of discussion themes

**Local Health Facility**

- Presence of a nurse, midwife, and/or doctor
- Availability of services for pregnant women (ANC, delivery etc)
- Availability of drugs and medical supplies
- Possibility of a blood transfusion and caesarean section (surgical operation)
- Accessibility from home; Quality of services; Affordability of services; Opening hours: before, during and after the conflict.
- Difficulties in accessing healthcare at the health facility level for MSRH services
- Factors affecting the use of local health facility for MSRH services
- Confidence in the local health system to cater for MSRH needs of women
- Impact of the conflict on the last 3 issues

Fourth set of discussion themes

**Impact of conflict on MSRH**

- Impact of conflict on maternal, sexual and reproductive health (with examples):

1. Individual
2. Health system (personnel, infrastructure, resources etc)
3. Accessibility to health services
4. Affordability
5. Quality

- Eyewitness accounts of specific negative consequences of conflict on MSRH
- Coping strategies developed to ameliorate the impact of the consequences of the conflict
