# Supplementary material for: A qualitative study exploring the determinants of maternal health service uptake in post-conflict Burundi and Northern Uganda
Source: BMC Pregnancy Childbirth. 2015 Feb 5;15:18. doi: 10.1186/s12884-015-0449-8 (PMC4327793; doi:10.1186/s12884-015-0449-8)
Supplement: Additional file 3: Table S2. — Factors affecting women’s utilisation of Maternal, Sexual and Reproductive Health Services (MSRHS) in post-conflict Burundi and Northern Uganda. This is a summary of the factors affecting women’s utilisation of maternal, sexual and reproductive health services in Burundi and Northern Uganda as perceived by women of reproductive age, local health providers and staff of NGOs working in the domain of maternal and reproductive health. The factors are further classified into individual level factors, socio-cultural level factors and political and health system level factors. [file 12884_2015_449_MOESM3_ESM.docx]

**Additional file 3: Table 2: Factors affecting women’s utilization of Maternal, Sexual and Reproductive Health Services (MSRHS) in post-conflict Burundi and Northern Uganda**

| **Participant category** | **Factors affecting women’s utilization of MSRHS** | | |
| --- | --- | --- | --- |
|  | **Individual** | **Socio-cultural** | **Political and Health system** |
| **Northern Uganda** |  |  |  |
| Women | -Development of a ‘danger sign’ during pregnancy  -Severity of the danger sign  -Previous loss of a child during delivery  -Burden of household chores/ availability of ‘free time’  -Previous bad experience with contraceptive use (severe side effects)  -Fear of confronting the ‘bad’ health provider at the facility  -Difficulties with caring for additional children  -Desire to recuperate after delivery (for uptake of contraceptives)  -Literacy/educational level | -Poor road network  -Uncertainty about HIV status  -Proximity to health facility  - Rumours and myths about contraceptives  -Level of support from immediate family during pregnancy / lack of assistance with household chores  -Ignorance  -‘Stupidity and stubbornness’  -Fear of being diagnosed with HIV  -Economic pressures on limited resources/ need to plan family size  -Male-partner opposition to contraceptive use and VCT services  -Access to transport (means of transport) to facility  -Cultural perception of women on contraceptives as being like ‘men’ (loss of womanhood)  -Fear of caesarean section  - Fear of side effects of contraceptives | -Universal health care policy  -Community delivery of services  - Attitude of personnel towards clients/ manner of reception of clients at the facility  -Delivery of services at the community level via mobile outreaches  -Physical presence of personnel at the facility  -Policy on male-partner companion for ANC visits and other services  - Construction of more health facilities  - Policy of providing of incentives to pregnant women such as delivery kits, mosquito nets etc. |
| Health Providers and NGOs | - Trust that confidentiality and privacy shall be respected  -Fear of confronting the ‘bad’ health provider at the facility  -Availability of a contraceptive method that can be concealed from partner  -Previous experience with delivery complication(s)  -Personal struggles to cater for the needs of many children  -Availability of ‘helping hands’ at home in their absence  -Previous bad experience in the hands of a health worker | -Economic pressures on limited household resources/ need to plan family  -Poor transport network  -Strong respect for TBAs in some areas / availability of TBA to assist with home delivery  - Proximity to health facility  -Weak family social and financial support systems for women  -Literacy level/ignorance  -Cultural perception of pregnancy as a normal condition  -Rumours and myths about contraceptives  -Male-partner support/non-support of contraceptive use  -Perceived side effects of contraceptives  -Desire to space deliveries and reduce pregnancy-related complications  -Desire to replace lost family members from the conflict  -Perception among some men that women on contraceptives are ‘stubborn’ and promiscuous  -Cultural desire for large family size  -Strong influence of the catholic church against the use of modern contraceptives | - Universal health care policy  -TBA abolition policy  -Delivery of some services at the community level  -Increased level of sensitization on radio  -Improved availability of services via mobile outreach clinics  -Requirement for women to guarantee partner’s support before enrolling for a contraceptive method  -Availability of youth-focused- and -friendly services  -Policy to provide feeding support to HIV positive mothers at the facility  -Free anti-retroviral therapy policy  -Coverage of delivery-related expenses by local politicians at private health facilities  -Acute shortage of qualified health personnel  -Attitude of health personnel  -Conduct of personnel with respect to privacy and confidentiality  -Regular stock-out of medicines and other supplies/ availability of medical supplies/ the ‘push’ drug policy  -Poor coordination between health institutions, NGOs and district health offices  -Effective integration of services  -Effective client follow-up for HIV-related services  -Quality of services  -Availability of incentives for women during pregnancy and delivery  -Poor management of teenage mothers/ Poor attitude towards teenage mothers |
| **Burundi** |  |  |  |
| Women | -Fear of developing complications during pregnancy and delivery  -Need to know HIV status  -Secure birth notification document to facilitate obtaining a birth certificate  -Fear of being diagnosed with HIV  -Difficulties with caring for additional children  -Literacy level  -Previous experience of a poor pregnancy and/or delivery outcome  -Previous poor outcome (side effect) with contraceptive use  -Previous bad encounter with personnel at the facility  -Fear that children born at home will not be registered  -Desire to commence family planning  -Desire to know the evolution of the pregnancy  -Lack of money/poverty  -‘Lack of good clothes’  -Lack of clothes for the newborn | -Poverty  - Proximity to health facility  -Male partner non-support of contraceptive use  -Lack of land for a large family  -Side effects of contraceptives  -Rumours and myths about contraceptives | -Free healthcare for pregnant women and children under five years  -Increased community-level sensitization  - Construction of more health facilities  -Recruitment of more health personnel  -Attitude of health personnel |
| Health Providers and NGOs | -Desire of HIV positive pregnant women to protect their unborn children from HIV  -Acknowledgement of the importance of family planning  -Realization on the importance of the services  -Desire to commence family planning  -Desire to understand pregnancy evolution  -Previous positive experience with contraceptive use  - Personal religious convictions  -Confidence in TBAs to conduct deliveries(presence of an alternative to facility-delivery)  -Desire to get birth notification document from hospital that will help in making a birth certificate for the baby  -Lack of money/poverty  -Ignorance | -Occasional financial costs  -Proximity to health facility  - Cultural practice of hiding a pregnancy during the first trimester  -Negative influence of religion/ Non-support of modern contraceptives by catholic church  - Limited acceptance of TBAs to undertake deliveries at home  -Growing desire for child spacing  -Rumours and myths about contraceptives  -Poverty  -Ignorance  -Male-partner opposition to contraceptive use  -Guarantee of personal safety/ improved security situation | - Free healthcare for pregnant women and children under five years  - Positive engagement with TBAs  -Use of locals for community sensitization  -Increased level and channels of community sensitization  -Use of facility issued birth notification document for issuance of birth certificate  -Introduction of performance-based financing(PBF) policy  -Provision of some contraceptive services at the community level  - Availability of competent personnel at the facility  -Wide range of services provided  -Availability of essential supplies at the facility  -Quality of services provided  - Promptness in the provision of services  -Attitude of personnel towards clients/ manner of reception of clients at the facility |
